# Biomaterials-based approaches to mandibular tissue engineering: where we were, where we are, where we are going

**DOI:** 10.1093/rb/rbaf024

**Published:** 2025-04-10

**Authors:** Maeve M Kennedy, Antonios G Mikos

**Affiliations:** Department of Bioengineering, Rice University, Houston, TX 77030, USA; Department of Bioengineering, Rice University, Houston, TX 77030, USA

**Keywords:** bone, 3D printing, bioceramics, scaffolds

## Abstract

The mandible is the largest craniofacial bone and plays a crucial role in speech, mastication, swallowing, and facial aesthetics. The form or function of the mandible can be altered by defects as a result of tumors, trauma, infection, and congenital conditions. This paper covers the evolution of biomaterials-based approaches to the reconstruction of critical size mandibular defects. Historically the gold standard for critical size mandibular defect repair has been autologous fibula grafts. The emergence of the field of tissue engineering has led to the current research on biomaterial scaffolds, cells, and biological factors to design highly tunable, bio-inspired, tissue regenerative implants. Scaffold materials can be synthetic or natural and can be fabricated using a variety of additive manufacturing techniques. Mesenchymal stem cells, bone morphogenetic proteins, and transforming growth factor-β are frequently added to scaffolds. While great progress has been made, there are still barriers to translating this research to patients, ranging from insufficient bone regeneration in animal studies to the feasibility of establishing a good manufacturing practice. To address these challenges, the future of mandibular tissue engineering will look toward improving implant vascularization and innervation, personalizing implant shape and biology, and enhancing spatiotemporal control of drug release. With these goals in mind, researchers will ultimately develop biomaterials that can regenerate bone that is structurally and biologically identical to native mandibular tissue, improving both function and quality of life for patients.

## Graphical abstract

**Figure:**
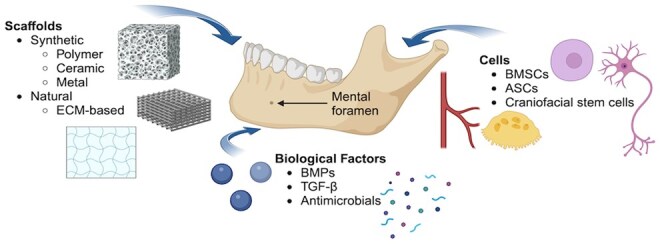

## Background: Mandibular Defect Causes and Impacts

Mandibular defects may arise from a variety of etiologies, including congenital conditions, malignant or benign tumor resection, osteoradionecrosis, osteomyelitis, and trauma [1]. The size of the defect is extremely impactful in the healing process and determines whether medical intervention is necessary. A bone defect is categorized as critical size when the native tissue is unable to bridge the gap between the remaining bone, and fibrotic tissue is deposited instead of mineralized bone tissue [2]. Below this critical defect size, the body’s inherent physiological healing processes can restore the damage, and therefore little to no medical intervention is required [3]. However, critical size defects necessitate medical intervention in order to enable healing.

The mandible is the single largest craniofacial bone and therefore plays a vital role in both function and aesthetics of the head and neck region. Functions including mastication, speech, swallowing, and airway accessibility all can be impacted as a result of mandibular defects. This not only decreases the quality of life for patients but also in more severe cases may increase morbidity and mortality [1, 4]. Additionally, people living with a craniofacial deformity can experience social stigma and psychological stress, such as depression and anxiety.

**Figure 1.**
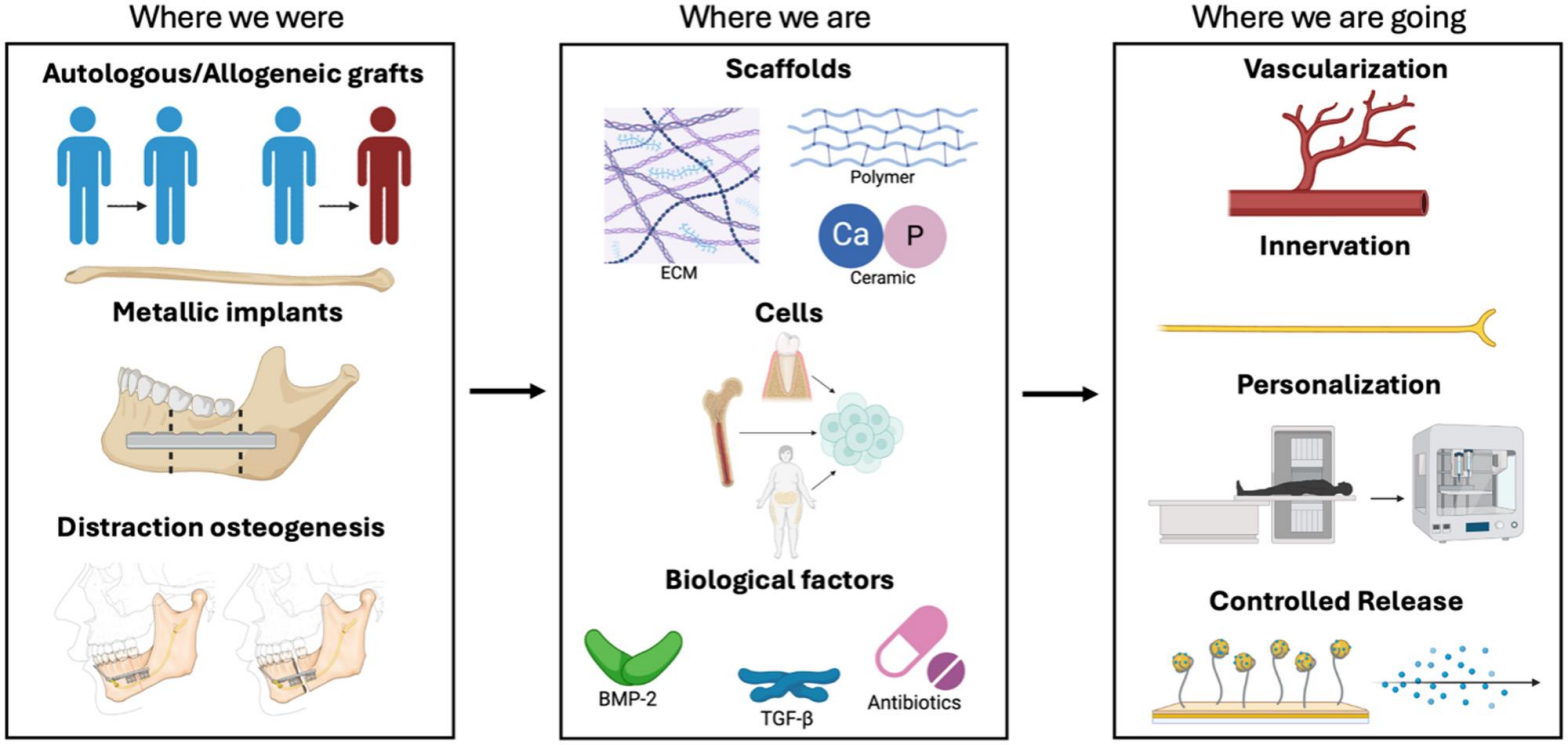
The progression of mandibular tissue engineering over time. In the past, bone grafts, metallic implants, and distraction osteogenesis were used. Currently, researchers are developing tunable implants consisting of a scaffold, cells, and biological factors to closely mimic the native mandible. In the future, it will be crucial to improve scaffold vascularization and innervation, personalize scaffold shape and components, and increase spatiotemporal control over biological factor release. Created using BioRender and image reproduced with permission from [5].

Given the significant impact of mandibular defects on a patient’s daily functions and quality of life, surgeons and engineers have worked together to develop reconstruction techniques. This review will outline the past, present, and future of mandibular defect repair, with a focus on tissue engineering and biomaterials (Figure 1).

## Where we were

**Figure 2.**
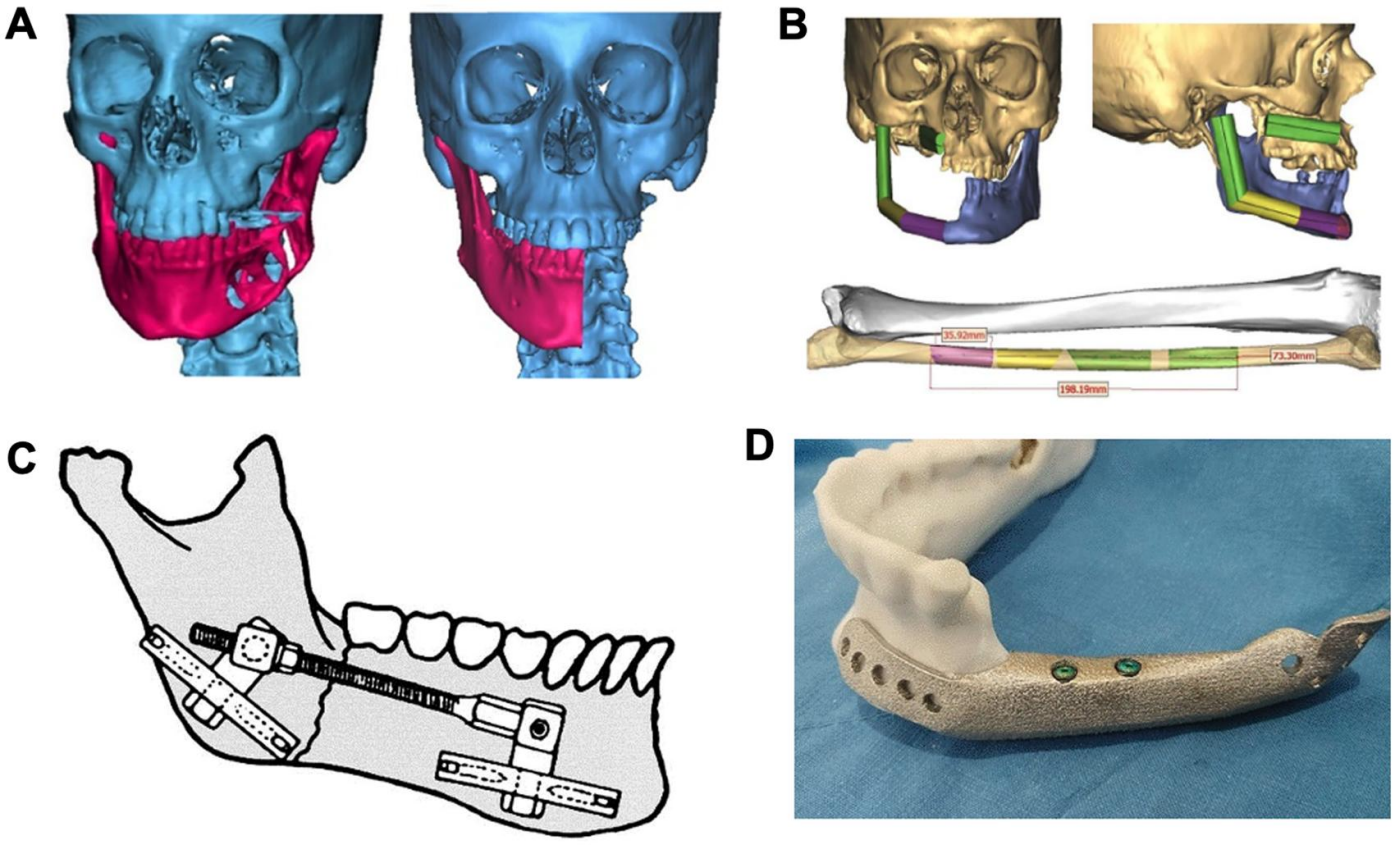
The history of mandibular defect repair. (**A**) Left: pre-operative 3D reconstruction of a patient’s mandible, demonstrating tumor invasion into the left lower jaw. Right: post-resection 3D reconstruction depicting the mandibular defect created by hemi-mandibulectomy procedure (reproduced with permission from [6]). (**B**) Fibular autograft harvest and transfer to reconstruct the right mandible and right maxillary alveolus (reproduced with permission from [7]). (**C**) Osteotomy creation in the lower jaw and subsequent distractor placement allow angular adjustments in two planes (reproduced with permission from [8]). (**D**) Surgical planning for attachment of titanium mandible implant plate to remaining jaw bone segment after resection (reproduced with permission from [9]).

Historically, the methods used for mandibular reconstruction have included autologous bone grafts, allogeneic bone grafts, metallic materials and distraction osteogenesis (Figure 2). While small mandibular defects can be adequately reconstructed using some combination of particulate bone, growth factors, and cells, the gold standard of care for critical size mandibular defects is an autologous bone graft from the fibula [10]. The fibula free flap is the most commonly used graft because its size and shape allow flexibility to shape the bone and the ability to procure large-diameter blood vessels [11]. Fibula flaps can either be vascularized or non-vascularized. It has been demonstrated that vascular flaps reduce recovery time and allow healing via tissue that is independent of the compromised recipient site [1, 4]. However, a limitation of the fibular flap is that the graft is limited by the volume and geometry of the donor tissue, and the amount of harvested material may be insufficient for large defects [12, 13]. In all autologous bone grafting, two separate surgical sites are required, thereby increasing the invasiveness and morbidity of the procedure [13]. Finally, no matter the choice of the donor site, there is a risk that graft integration with the native bone can fail. This can lead to tissue necrosis and removal of the graft, ultimately leaving the patient with the same if not worse defect. While autologous grafts can be customized for each patient, they are limited by the properties of the donor site and can have high associated morbidity.

Distraction osteogenesis was developed as a way to use the mandible’s own healing capacity to regenerate bone in the defect area. Briefly, a surgeon makes an osteotomy in the mandible and gradually separates the two bony surfaces over the span of 12–15 weeks depending on the length of the defect [14]. A fibrous callus forms on the ends of the injury site, which subsequently ossifies, and as they are pulled apart it slowly lengthens the new bone. Some surgeons may even prefer to make multiple points of distraction to facilitate faster bone regeneration. It is important to consider that many of these patients may be post-radiation or chemotherapy for a tumor involving the mandible. Radiation and chemotherapy affect the bone’s regenerative capacity and may lead to prolonged consolidation, so the timing of distraction osteogenesis is important [14, 15].

Another currently used method for mandibular defect repair is metal implants. During primary reconstruction of a defect, metal bridging plates can be used in the area lacking bone. Pure titanium is most often used because of its biocompatibility and high mechanical strength. Titanium alloys are less popular than pure titanium due to their lower biocompatibility; however, they do have higher mechanical properties. It is possible to produce patient-specific mandibular implants by selective laser sintering (SLS) of titanium [16]. A downside of metal implants is that fractures occur in 2.9–10.7% of cases, most often within 6 months of surgery [17].

While these methods have historically been the standard of care, in recent years, advancements have been made in developing biomaterials for mandibular reconstruction. The next section of this paper will explore the mandibular tissue engineering approaches that are currently under development.

## Where we are

The field of tissue engineering has emerged as an alternative approach to repairing mandibular defects. In general, the three pillars of tissue engineering are scaffolds, cells and biological factors. This review will discuss the current state of each of these pillars as they apply to mandibular tissue engineering.

## Scaffolds

Scaffolds should mimic both the micro and macro structure of native mandibular tissue. Two of the most important scaffold parameters are the pore size and porosity. Scaffolds should have interconnected pores to allow mass transport of oxygen and nutrients, which facilitate cell colonization. It has also been shown that there is an optimal pore size to encourage bone formation. Pores larger than 300 μm favor direct osteogenesis in conjunction with vascularization and high oxygenation; however, there is an upper limit because as pore size increases mechanical properties decrease [18]. It is crucial that these scaffolds are as close as possible to native mandibular tissue from a mechanical perspective in order to withstand the high mechanical loading of the jaw. The mandible consists of two types of bone: two outer layers of cortical bone and a thick inner layer of trabecular bone [19]. The trabecular bone in the human mandible has an average compressive modulus of 56 MPa and an average ultimate compressive strength of 3.9 MPa [20]. An added level of complexity is the fact that different parts of the mandible have different bone structures and mechanical properties, such as the symphysis compared to the ramus [19]. Zamani *et al*. designed a scaffold made of 3D printed poly(Ɛ-caprolactone) (PCL) with a gradient of pore sizes, and therefore a gradient of compressive and tensile strengths, in order to match the force distribution in the mandible [21]. Most tissue-engineered implants as of now cannot reach the mechanical properties of native mandibular tissue and cannot withstand the daily forces of the mandible.

### Synthetic materials

Scaffolds can be made of either synthetic or natural materials. Synthetic scaffolds include polymers, ceramics and metals. Synthetic polymers enable tuning of scaffold properties in a repeatable and reproducible manner. The polymer molecular weight, chemical composition, crystallinity and degradation rate are all important factors to take into account when selecting a scaffold material. Scaffolds for mandibular tissue engineering have been fabricated out of PCL, poly(lactic-co-glycolic acid) (PLGA) and poly(lactic acid) (PLA), among others [22–28].

While there are distinct advantages of synthetic polymers, the major drawbacks are that they have lower bioactivity, osteoconductivity, and cell adhesion sites. Ceramic–polymer composite scaffolds can help to overcome some of these drawbacks. Native bone is composed of approximately 40% organic components and 60% inorganic components [29]. The organic phase mostly consists of collagen type I and the inorganic phase mostly consist of calcium phosphate minerals. Tissue engineering scaffolds should have to have a similar composition to native bone tissue. Hydroxyapatite (HA), tricalcium phosphates (TCP) and biphasic calcium phosphates are the most studied mineral components in ceramics. HA is a calcium phosphate mineral and has structural characteristics similar to the inorganic components of bone. It is also osteoconductive and promotes osteoblast and osteocyte differentiation. HA is more stable, while TCP is more soluble. β-TCP has a faster degradation rate than HA due to its lower calcium/phosphate ratio [30]. The downside of ceramics is that they have low strength and high brittleness under tension, but this is overcome by combining them with polymers to create polymer–ceramic composites that can withstand more load bearing [30].

While permanent metal implants were one of the earliest technologies implemented for mandibular tissue engineering, the use of degradable metals has developed more recently. Magnesium and magnesium alloys are the most studied degradable metals for repair of mandible defects. Magnesium has a similar specific density and elastic modulus to bone, as well as degrades gradually in the body allowing space for new tissue infiltration [31]. Only a few studies have been reported, and most utilize bone screws, rods, or plates rather than segmental implants to repair critical size defects. However, these preliminary studies have shown that magnesium-based alloys enhance osteogenesis and osteoconductivity, promote an even distribution of stress forces on the mandible, and do not result in deleterious effects from local degradation products [32]. Limitations to biodegradable magnesium implants include premature degradation, inhomogeneous degradation, and potential concern from the release of hydrogen gas as a result of degradation [31].

### Natural materials

Natural scaffold materials are those derived from native tissue and extracellular matrix (ECM) components, which can provide more meaningful cues to cells compared to synthetic materials alone. ECM-based materials have shown promise as a biomaterial for tissue engineering given their complexity, biocompatibility and mimicry of a microenvironment conducive to tissue regeneration [33]. The bone ECM is synthesized by osteoblasts and consists of type I collagen, glycoproteins, proteoglycans and growth factors [34]. ECM can be incorporated into scaffolds as individual components or as the entire ECM derived from a decellularization process.

ECM-based materials have been applied to mandible regeneration with success. In a 2017 study performed in human patients, a decellularized lyophilized bone graft was used to reconstruct mandibular bone defects post oncologic resection [35]. This study showed that the ECM structures remained intact after lyophilization and prevented fibrous tissue invasion. Demineralized bone matrix nanoparticles have been incorporated into colloidal inks for 3D-printed osteoinductive and osteoconductive scaffolds for craniofacial bone repair [36]. Another method of incorporating ECM is simply coating the scaffold. Lee *et al*. created a PCL/TCP scaffold by fused deposition modeling (FDM) and then coated it in demineralized decellularized bone ECM to create a microenvironment that directs long-term cell proliferation and differentiation [22].

There are many existing techniques used to fabricate tissue engineering scaffolds, whether they are made of synthetic or natural materials. This review will describe additive manufacturing, electrospinning and bioreactors, as these are the most commonly used in current tissue engineering research for the mandible.

### Additive manufacturing

**Figure 3.**
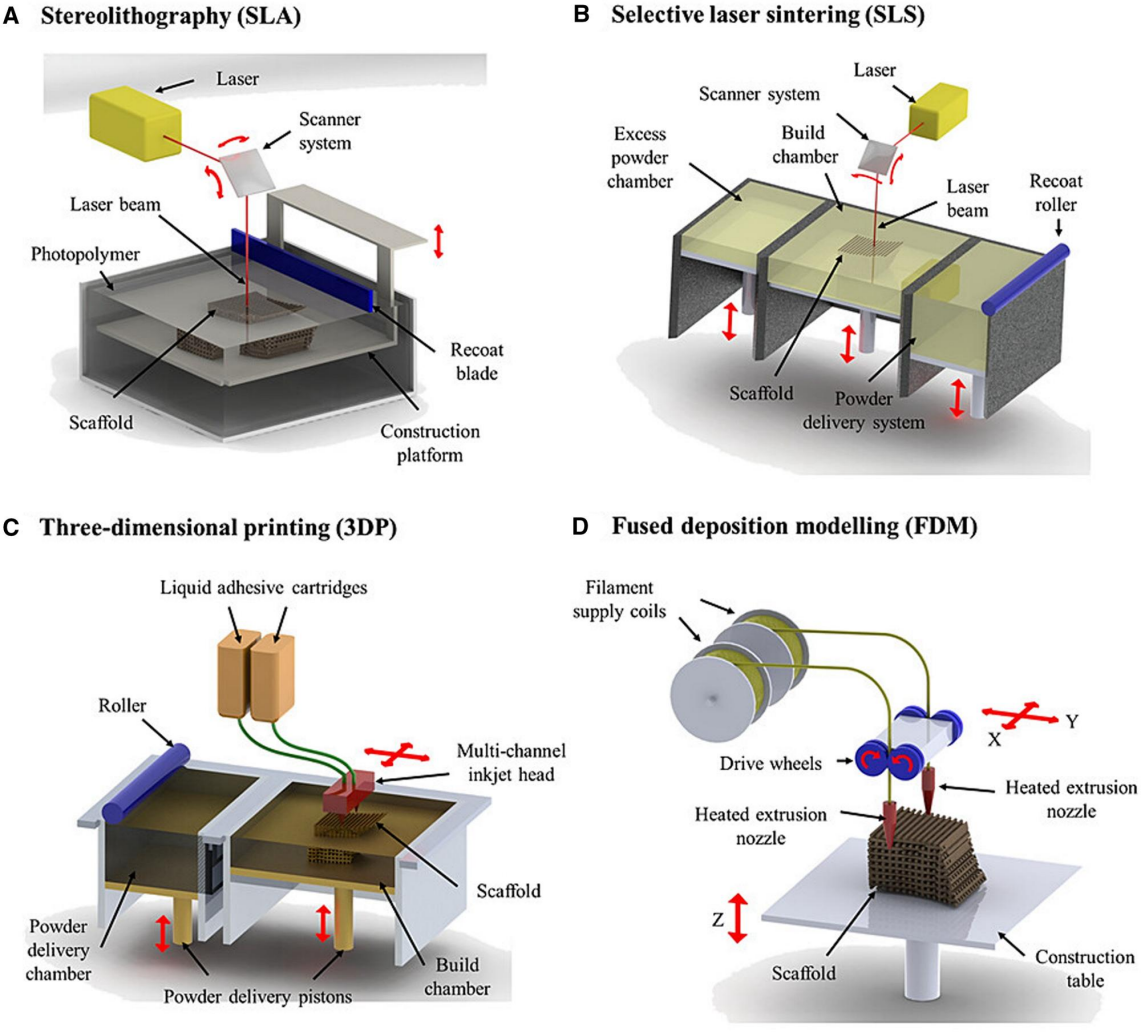
Additive manufacturing techniques are commonly used for mandibular scaffold fabrication (reproduced with permission from [37]). (**A**) Stereolithography utilizes a photocurable resin, (**B**) selective laser sintering employs a laser to fuse powdered material into a solid, (**C**) 3D extrusion printing forces a thermoplastic material through a heated nozzle for layer-by-layer deposition, and (**D**) fused deposition modeling extrudes a heated thermoplastic filament layer-by-layer.

Additive manufacturing is the process of building a 3D construct by adding material layer-by-layer [37]. It allows the creation of porous scaffolds with precise control of complex geometries in order to mimic the microarchitecture of the mandible, all in a relatively easy to learn, low-cost and accessible manner [19]. Additive manufacturing is the most commonly used technique for fabricating scaffolds for mandibular tissue engineering. The different types of additive manufacturing techniques include melt extrusion, FDM, stereolithography and SLS (Figure 3). Ceramics, polymers and metals can all be used as materials in additive manufacturing.

Melt extrusion is the process of using a pressurized gas, pressurized piston, or screw to flow molten material through a nozzle and deposit it layer-by-layer [38]. Its ability to control scaffold geometry, filament diameter and filament orientation makes it incredibly useful for tissue engineering. Melt extrusion has been successfully used with hydrogel and thermoplastic polymer inks. Thermoplastic polymers require solvents or high temperatures to extrude, so cells cannot be added directly to the printing ink [38]. Extrusion 3D printing can be used to make large-sized models at a low cost. Limitations to melt extrusion are difficulty achieving fine structures, high printing temperatures and weak adhesion at layer interfaces. Many papers have been published that use melt extrusion to fabricate scaffolds for mandibular tissue engineering, often using a patient-specific computed tomography (CT) scan as the scaffold design [19, 21].

Melt electrowriting is another additive manufacturing method and entails a heated polymer that is exposed to a high-voltage electric field as it flows out of a nozzle toward the collector [39]. Its advantages over melt extrusion include the precise deposition of micro- and nano-fibers and ability to create nonlinear scaffold structures [39]. There is not much literature on melt electrowriting for the mandible specifically, but melt electrowritten PCL scaffolds coated in collagen and fluorinated-calcium phosphate have been shown to significantly increase osteocalcin expression, indicating osteogenic potential for craniofacial bone regeneration in general [40].

FDM involves heating a thermoplastic filament to a semi-liquid state, and then extruding it through a nozzle [41]. Compatible polymers have thermal stability, flow qualities and mechanical resistance [41]. FDM takes place between approximately 90°C and 450°C depending on the polymer being used, therefore any biological components need to be added post-printing. A major drawback of FDM is limitations in commercially available filaments, and the high cost and effort associated with designing a custom filament. There are several examples of FDM being used to fabricate mandibular tissue engineering scaffolds. Addressing the previously mentioned challenge of incorporating biological factors, Bouyer *et al*. first used FDM to print a PLA porous scaffold at 190°C and subsequently coated them in poly(L-lysine) and hyaluronic acid to render them osteoinductive before implantation into a critical-sized mandibular defect *in vivo* [27]. Another group printed PCL + β-TCP at 120°C, which were then coated in collagen and recombination human bone morphogenetic protein 2 (rhBMP-2). FDM allowed them to create a structurally complex implant, with a cancellous bone-like layer and a cortical bone-like layer, consisting of varying pore sizes including 100, 300 and 400 μm [23].

Stereolithography utilizes photopolymerization via laser/blue light/UV to cure a liquid resin. This method offers high-resolution printing and fast processing times, but there is limited availability of biocompatible resins, which can make clinical translation more difficult [19]. Resins such as PLA, PLGA and poly(propylene fumarate) with reactive acrylate or methacrylate can cause cytotoxicity and inflammatory reaction from their macromer precursor, reactive moieties or degradation products [42]. Photopolymers, photoinitiators, dye-initiators and solvents can all cause cytotoxicity, but this can be somewhat circumvented by adding well established scaffold materials, such has HA, to the photopolymers [43]. Drawbacks of stereolithography include scaffold shrinkage during post-processing and the necessity of support structures to enable printing of complex shapes [37]. Stereolithography has previously been used in mandibular defect applications. For example, a resin loaded with HA and TCP was cured with a digital light processing 3D printer and used to print porous scaffolds that were placed in a rectangular lower jaw defect in beagles [44]. In another study, jawbone-derived osteoblasts were added to a gelatin methacrylate (GelMA) and hyaluronic acid ink and printed using stereolithography. Culture of these human jawbone models led to differentiation of osteoblasts to osteocytes and mineralization [45].

SLS is the process of selective sintering of a polymer powder using a laser beam, and then depositing subsequent layers of powder on top and repeating the lasering to build the object layer-by-layer [37]. The laser is capable of forming bonds both between individual particles in a layer, and between adjacent layers, for maximum cohesion. Scaffolds can be produced by SLS rather quickly and do not require any support structures; however, they generally have low mechanical properties [37]. SLS is less commonly used to create scaffolds for mandibular tissue engineering; however, it has been done before. A composite ceramic implant made of poly(DL-lactic acid) (PDLLA) and β-TCP was implanted in a critical size defect in the jaw of rats. Using PDLLA allowed the composite to melt layer by layer and therefore print at temperatures that would not alter the structure of β-TCP [28]. Another instance of the utility of SLS for mandibular tissue engineering was Li *et al*.’s fabrication of a titanium grid scaffold with gradient pore sizes that was implanted in critical size defects in human mandibles as part of a clinical trial. Their grids had over 90% porosity which is difficult to print, but SLS allowed it to be possible [16].

Bioceramic materials have shown great value for mandibular tissue engineering and can be fabricated using additive manufacturing techniques. A ceramic composed of 1:1 PCL:β-TCP made using FDM was seeded with tonsil-derived mesenchymal stem cells and implanted into a mandible defect model [46]. Another example was 70% PCL 30% β-TCP printed using a micro extrusion-based 3D printer to make personalized mandible implants based on canine CT scans [47]. Bioceramics have also been printed with stereolithography, specifically a crosslinked network of poly(trimethylene carbonate) and β-TCP for patient-specific mandible bioimplants [48]. Finally, Lopez *et al*. showed that a 100% β-TCP colloidal gel could be printed with a 3D direct-wrote micro printer gantry system to create porous scaffolds for rabbit mandible critical size defect repair [49].

In summary, there are many additive manufacturing techniques available for fabricating bone tissue engineering scaffolds for the mandible. Those described in this review are the most commonly used, however, less frequently used alternative techniques exist. Each technique has its own advantages and drawbacks, which can inform the user’s choice of additive manufacturing method.

### Electrospinning

Electrospinning is a technique to create micro- or nano-scale fibers that represent the 3D environment of the ECM. Briefly, a liquid polymer or hydrogel is subjected to a high-voltage electric field, which propels the solution to a collection plate as the solvent evaporates, depositing a mesh of interconnected fibers. This 3D structure guides cell morphology and creates an environment more conducive to cell proliferation and differentiation. This is beneficial both for *in vitro* cell culture work and *in vivo* scaffold implantation for tissue repair and regeneration. The pore size, porosity, fiber diameter and fiber alignment are highly tunable by varying the electrospinning conditions. There is also great flexibility in the choice of electrospun fiber material, such as gelatin, silk, collagen, chitosan, hyaluronic acid, synthetic polymers, inorganic materials and even cells or other biologic materials.

Electrospun scaffolds have weak mechanical properties, and so are not usually used on their own for mandibular tissue engineering purposes. They have, however, been combined with more robust scaffolds to fulfill both mechanical and biological requirements. For example, an alternating structure of 3D printed and electrospun PCL was prepared and preosteoblasts were cultured on the scaffold. The scaffold showed a compressive modulus of up to 0.35 MPa and facilitated osteogenesis *in vitro* [50]. In another study, Hany *et al*. fabricated an electrospun PCL scaffold coated in alginate and nano HA, which was then placed in a rabbit mandible defect model. After 5 weeks the defect site contained cartilage, woven bone and lamellar bone at both the margins and the center [51].

Electrospinning offers a promising addition to ECM-inspired scaffolds that are highly tunable. However, their poor mechanical properties require additional support structures for mandibular bone applications.

### *In vivo* bioreactors

**Figure 4.**
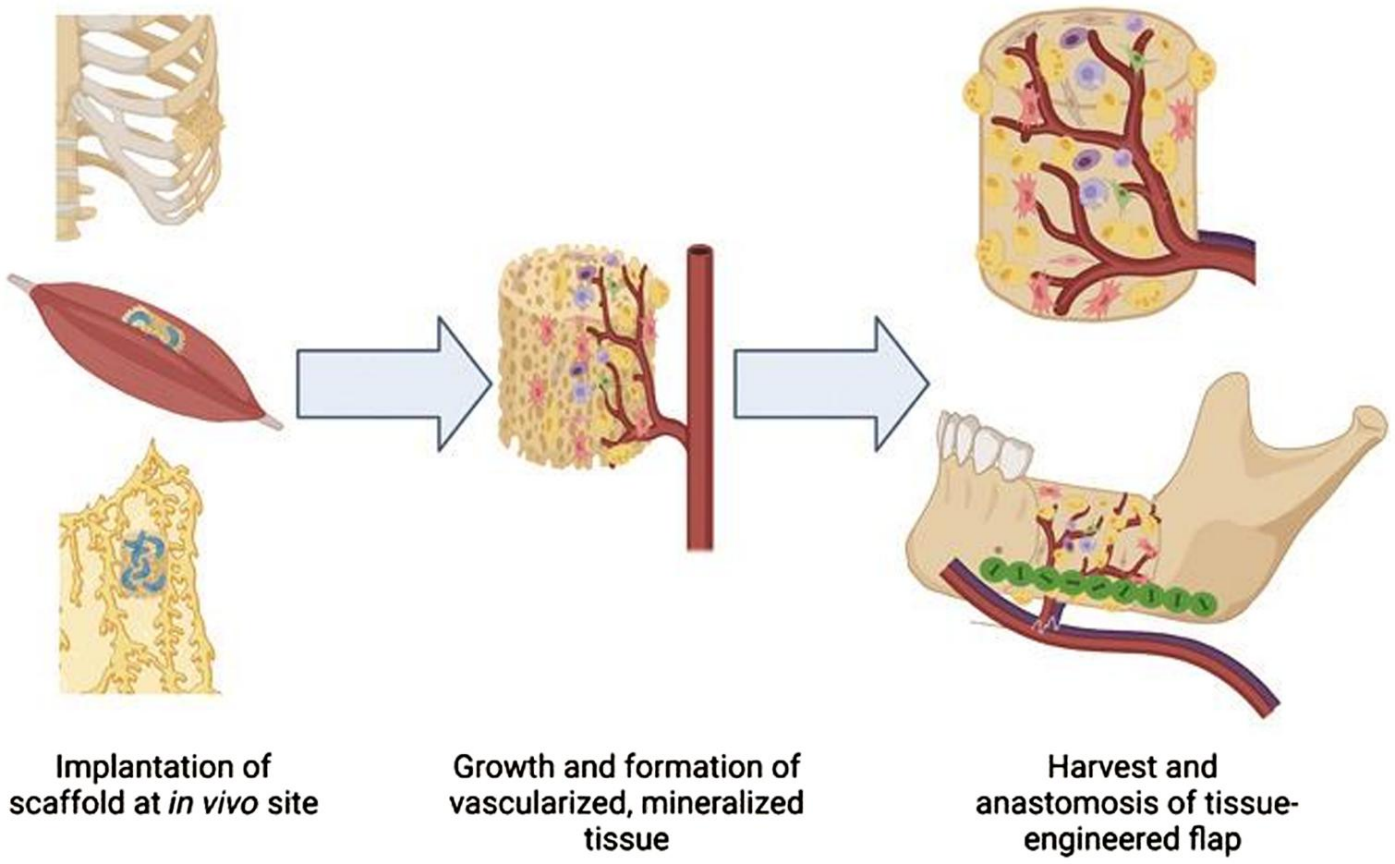
The strategy for bone regeneration based on *in vivo* bioreactors involves scaffold implantation adjacent to rib periosteum or intramuscularly or in the omentum, several weeks of cell migration to the scaffold leading to capillary formation, and finally transfer to the mandibular defect site with adjacent vasculature (reproduced with permission from [29]).

One of the newer methods for mandible scaffold fabrication implements an *in vivo* bioreactor. In this technique, the scaffold is implanted at a site ectopic to the defect and left there to mature and generate mineralized tissue for some time before being transplanted to the mandible (Figure 4). The bioreactor can be filled with osteoconductive material which stimulates the native cell populations to migrate to the bioreactor and generate new bone [12]. This method is especially advantageous for the mandible and other craniofacial tissue engineering when the defect site is infected or there is a risk of stimulation of cancerous cells with growth factors, and reconstruction cannot be performed immediately [29]. If only bone regeneration is desired, the periosteum is an ideal bioreactor site. However, if vascularization is also desired, then muscle or omentum is an effective bioreactor site [29]. Drawbacks to this technique include the inability to monitor the mineralization of the scaffold while it is in the *in vivo* bioreactor, the patient burden from undergoing two procedures, and the reliance on the patient’s endogenous regenerative capacity [29].

For the mandible specifically, Tatara *et al*. created a 3D-printed porous bone space maintainer made of poly(methyl methacrylate) and filled with autologous bone, which was implanted next to the rib periosteum for 9 weeks and then grafted into a mandibular defect as a free flap [12]. This promoted the formation of mature bone tissue with mechanical properties similar to those of the native mandible. Building upon this, Watson *et al*. created a mandibular defect model with localized Staphylococcus aureus infection and simultaneously implanted *in vivo* bioreactors containing autograft bone next to the rib periosteum of sheep. They found that the presence of local infection of the mandible increased the density and maturity of bone formed in the distal bioreactor, indicating the role of bacterial osteomyelitis-related pro-inflammatory cytokines in fracture healing and bone formation [52].

## Cells

In order to promote and expedite cellular infiltration and tissue regeneration within scaffolds, cells are frequently seeded on scaffolds prior to implantation. Stem cells are the most studied in mandibular tissue engineering due to their ease of attainment and expansive differentiation capabilities. A variety of stem cell origins have been studied in mandibular tissue engineering scaffolds.

Given that the mandible is made of bone, bone-derived stem cells are the most widely used cell type for this application. It is important to keep in mind the embryonic origin of the mandible when selecting a bone stem cell origin. The mandible is derived from neural crest cells, and it is known that bone marrow-derived stem cells (BMSCs) may have site specific preferences and behaviors depending on their embryonic origin [53]. This was proven in a study where mesoderm-derived progenitor cells failed to differentiate into osteoblasts when implanted in a mandible defect, but were able to facilitate normal repair when implanted in a tibia defect [54]. This finding suggests that choosing stem cells derived from the same origin as the mandible is beneficial. An example of successful use of BMSCs is from a study where BMSCs were seeded onto a 3D-printed PCL/β-TCP scaffold and implanted in a minipig mandibular defect model. The addition of cells caused deeper bone penetration depth and increased CD31 expression compared to the acellular groups [24].

Although BMSCs have greater osteogenic capacity compared to other stem cell types, the process of harvesting them from the iliac crest or sternum can be very painful for patients and can yield a low amount of cells [55]. While studies have shown that a posterior approach to the ilium can increase cell yield and decrease postoperative pain, other sources of mesenchymal stem cells can still be more advantageous [56]. Adipose-derived stem cells (ASCs) are easier and safer to acquire than BMSCs making them a viable alternative cell source. ASCs are harvested from any adipose tissue in the body, most common being subcutaneous adipose tissue in the abdomen, thigh, or arm. ASCs have been used in mandibular defect models. For example, Lee *et al*. added ASC aggregates to a PCL/TCP scaffold and found that the addition of ASCs raised the expression of osteogenesis-related genes encoding collagen 1, osteocalcin and Runx2 [24].

Moving to more craniofacial-specific cells, stem cells from deciduous teeth have previously been seeded onto β-TCP scaffolds and placed into critical size mandibular defects in minipigs. The deciduous teeth stem cells were found to have differentiated into new bone, with more mineralized matrix, new islands of bone and newly formed blood vessels [57]. Another stem cell source is dental pulp stem cells, which Zhang *et al*. seeded on a polycarbonate/β-TCP scaffold that was implanted into a critical size defect in the lower jaw of rabbits. The implant showed more robust and homogeneous bone formation that represented native bone, with active remodeling and a high degree of vascularization [58]. A third option is sourcing MSCs from gingival tissues, which are from neural crest origin and have demonstrated osteogenic differentiation potential in craniofacial applications [59]. Finally, a different publication incorporated periodontal ligament stem cells into a lyophilized nano HA/chitosan/gelatin scaffold. When implanted into a critical size defect in the jaw of minipigs, there was significantly increased bone formation and increased expression of Runx2, a key transcription factor in osteoblast differentiation [60].

While the current focus is on pre-seeding cells and culturing them on the scaffold prior to implantation for mandibular regeneration, there are a few drawbacks to this approach. This method requires a significant amount of equipment, such as bioreactors, biosafety cabinets and incubators, as well as specialized staff trained in cell culture [19]. Looking to the future of mandibular tissue engineering, it would be ideal for the material itself to stimulate the body’s own regenerative potential and stimulate the pre-existing cells in the defect.

## Biological factors

While tissue-engineered scaffolds are excellent for replicating the structural properties of mandibular bone, they have limited cell adhesion and bioactivity. Combining scaffolds with biological molecules, growth factors and drugs can enhance the biological activity of implants. An advantage of biological factors compared to cells is that they do not require the time and resources needed for cell culture, and because of this they can be manufactured more quickly. However, growth factors have short half-lives, so their effects are not long-lasting if simply added to a defect without any coexisting carrier material. To extend their time of efficacy, biological molecules can be directly incorporated into the scaffold material, coated on the scaffold surface, or encapsulated in microcarriers within the scaffold.

One of the most commonly used growth factors in mandibular tissue engineering are BMPs. BMPs are secreted by osteoblasts and promote ossification by stimulating differentiation of mesenchymal stem cells into osteoblasts and the proliferation of osteoblasts and chondrocytes [27]. For example, a study placed PLA implants coated with a polyelectrolyte film delivering BMP-2 into a critical size minipig mandibular defect, and demonstrated a clear dose-dependence on BMP-2 for the amount of regenerated bone and repair kinetics [27]. Therefore, a controlled release strategy is desirable. Using a PLGA/nano HA scaffold, Deng *et al*. found that the incorporation of chitosan/rhBMP-2 nanocarriers had osteogenic effects resulting in a higher bone volume fraction in a mandibular defect model [25]. Additionally, the use of nanocarriers was able to control for the early release burst effect. The biggest concern when using BMPs in craniofacial surgery is the risk of orofacial swelling [61].

Transforming growth factor beta (TGF-β) is another growth factor that plays a role in osteoblast and BMSC proliferation and differentiation, as well as ECM interaction during osteogenesis. Hence, TGF-β may be combined with tissue engineering technologies to modulate the cellular environment during bone regeneration. TGF-β has been used in scaffolds for mandibular defect repair in animal models with success. In an early study, a mixture of gelatin and 0.1 µg of TGF-β1 was lyophilized and scaffolds were added to mandibular defects of rats. The addition of TGF-β1 significantly increased the degree of defect closure and the amount of newly formed mineralized bone, presumably due to the prolonged release of TGF-β1 from the hydrogel over the course of 6 weeks [62]. In a different study, lyophilized silk fibroin-chitosan scaffolds were loaded with 100 ng of TGF-β1 within the scaffold. *In vivo*, scaffolds were inserted into lower jaw defects in rabbits for up to 12 weeks. Results showed full closure of the defect with osseointegration between the implant and native bone, increased bone mineral density and increased scaffold flexural strength [63].

Antimicrobials are another powerful biological factor that has been incorporated into mandibular scaffolds. Mandibular defects are inherently prone to infection given their location in the mouth and the exposure to oral flora. The abundant blood supply to the mandible and its irregular complex shape also make it more prone to infection [64]. To prevent infection, researchers have developed scaffolds with antimicrobials incorporated to offer localized and sustained release. Zhang *et al*. 3D printed a multi-material scaffold consisting of PLGA, TCP, chlorhexidine and graphene oxide. When implanted into a critical size mandibular defect that had been seeded with *Staphylococcus aureus*, after 1 week there were no colonies formed from the implant surface and a low amount of inflammatory tissue surrounding the implant [64].

While the tissue engineering dogma of scaffold, cells and biological factors has led to more bio-inspired implant designs for mandibular repair, there are still challenges facing the field ranging from the properties of the mandible itself to the feasibility of clinical translation.

## Current challenges in tissue engineering the mandible

There are several challenges currently facing the field of mandibular tissue engineering. First, due to physiologic differences, it is not scientifically accurate to translate long bone tissue engineering research to mandibular tissue engineering research. The mandible heals through intramembranous ossification rather than endochondral ossification, and stem cells in the mandible have higher osteogenic potential compared to other skeletal bones [65]. Maxillofacial bones also have a higher remodeling rate, but a lower degree of mineralization and mass density compared to the femur [66]. Most current bone tissue engineering research focuses on long bones, but these key physiologic differences emphasize the fact that there is a need for research that is specific to the mandible. Another challenge in this field is the lack of data from clinical studies. There is a paucity of clinical studies on biomaterials for replacing mandibular defects, and the currently available studies have inadequate follow-up times, small sample sizes, and sometimes a lack of control groups [67]. Most are case reports with relatively low evidence value, underscoring the urgent need for more quality clinical studies of mandibular tissue engineering constructs.

## Clinical translation of tissue engineering approaches to mandible regeneration

An overview of clinical studies applying tissue engineering to mandibular defect repair is included in Table 1. While there has been significant progress made in research for this area, the clinical application lags far behind. Therefore, most of these clinical trials were scaffold-free or used a metal implant, which are some of the simpler approaches. If the previously mentioned challenges can be addressed, more robust clinical trial data can be generated and lead to tissue engineering scaffolds becoming more commonly used for mandible defect repair.

**Table 1.** Clinical translation of tissue engineering approached for mandible defect repair in humans

Author, year, reference	Sample size	Defect cause	Tissue engineering approach	Follow-up time	Outcome
Chen *et al*., 2024 [68]	7	Tumor resection	Beam additive manufacturing of titanium alloy implant	6 months	2/7 had implant exposure and required second surgery
Li *et al*., 2023 [16]	3	Tumor resection	3D-printed titanium implant	6–36 months	Successful function and aesthetics; mineralization and vascularization in new bone area
Zhong *et al*., 2022 [69]	2	Mixed (benign and malignant tumor resection)	Selective laser melting of titanium implant	6 months	No complications
Li *et al*., 2022 [70]	6	Mixed (tumor resection, osteomyelitis)	3D-printed PEEK implant	10–24 months	5/6 had successful function and aesthetics; 1/6 had implant exposure
Melville *et al*., 2020 [10]	34	Mixed (benign tumor resection, trauma)	Allogeneic bone + BMP-2 + bone marrow aspirate concentrate	6 months	90% achieved bony union
Gjerde *et al*., 2018 [71]	11	Tooth loss	Autologous BMSCs + biphasic calcium phosphate injectable	12 months	No adverse effects, increase in total bone volume, formation of mineralized tissues, successful function and aesthetics
Qassemyar *et al*., 2017 [72]	2	Tumor resection	SLS of titanium implant	12–18 months	Successful function and aesthetics
Rachmiel *et al*., 2017 [73]	1	Tumor resection	SLS of titanium implant + autologous bone graft + bone xenograft	12 months	Successful function and aesthetics
Leiser *et al*., 2016 [74]	1	Trauma	SLS of titanium implant + autologous bone graft	6 months	Successful function and aesthetics
Shan *et al*., 2015 [75]	2	Tumor resection	FDM of titanium alloy implant + autologous bone graft	6 months–5 years	No complications
Ferretti and Ripamonti, 2002 [76]	6	Mixed (tumor resection, trauma)	Demineralized bone matrix + BMP paste	12 months	2/6 had new bone formation with mineralization and trabeculae

## Where we are going

While significant progress has been made in mandibular tissue engineering, there are still barriers remaining to translating this technology to use in patient care. Biomaterial scaffolds have had limited clinical use for mandible repair due to insufficient amounts of bone regeneration, complications, or a lack of long-term stability [77]. These challenges are directing the field toward the future of mandibular tissue engineering. The most exciting areas for future mandibular tissue engineering are vascularization, innervation, personalization, and controlled release.

## Vascularization

Current literature suggests that adequate vascularization may be the largest bottleneck in mandibular tissue engineering. Endothelial cells secrete a variety of growth factors that “regulate the recruitment, proliferation, differentiation, function and survival of osteoblasts and osteoclasts” [78]. As a result, the regeneration of bone tissue is not possible without surrounding blood vessels nearby. There are three cutting-edge approaches to enhancing the vascularization of scaffolds for mandibular regeneration; however, they all require more refinement before their use in patients.

The first approach is controlling the scaffold geometry for optimal blood vessel formation. The pore size, porosity, and pore interconnectivity influence the ability of blood vessels to infiltrate scaffolds. In a study by Qin *et al*., it was determined that a 600-μm pore size was most suitable for new bone growth based on the presence of major arteries and high-density neovascularization in a mandibular defect model [79]. A smaller 480-μm pore and larger 720-μm pore had a higher number of blood vessels, but they were immature. This can be explained by the fact that small pores do not allow for nutrient and oxygen supply and therefore osteogenesis, while large pores can lead to saturation of nutrient and oxygen supply as well as allow more room for undesirable fibrous tissue penetration [79].

**Figure 5.**
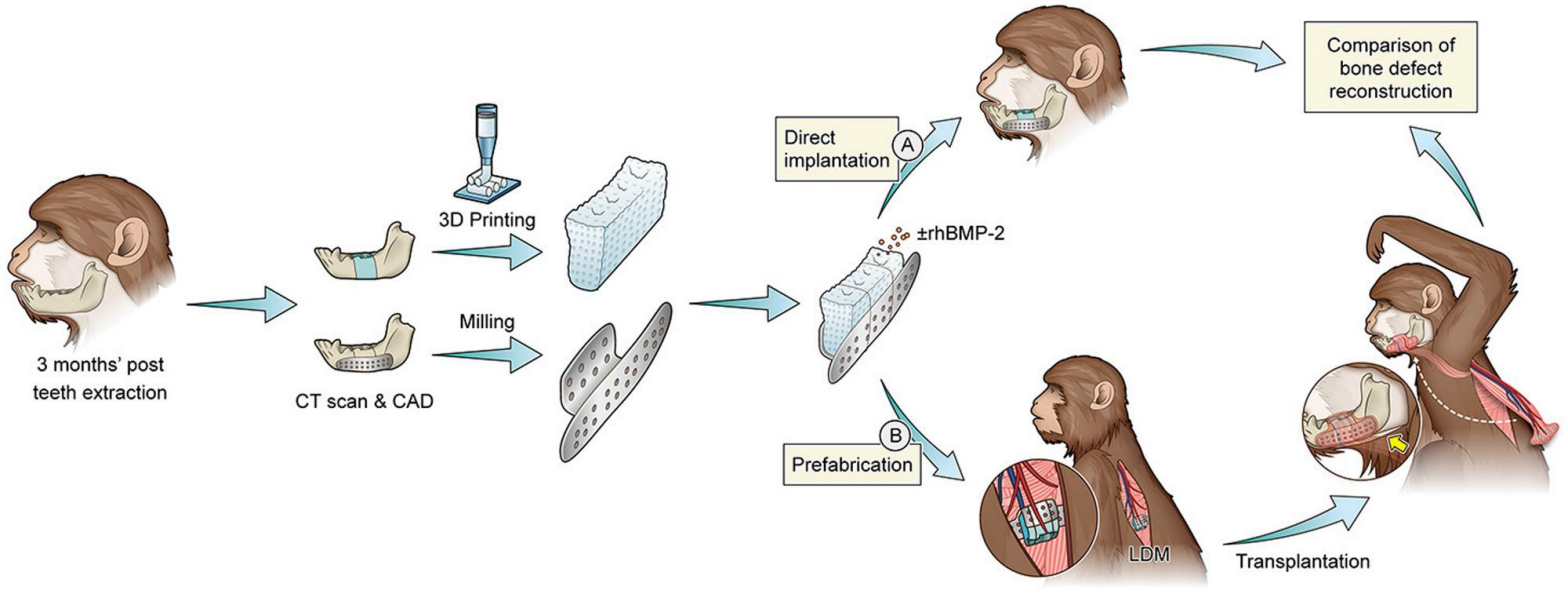
Custom implants made of titanium mesh and 3D-printed PLGA/β-TCP scaffolds were coated in rhBMP-2 and implanted into the latissimus dorsi muscle of a primate to allow for prevascularization prior to implantation in a mandibular defect (reproduced with permission from [26]).

The second approach is using a living bioreactor to pre-vascularize the scaffold. The scaffold is first implanted into a highly vascularized site in the body, for example, skeletal muscle, and left for several weeks to allow blood vessel formation. Then the scaffold is grafted to the recipient site and attached to the local blood supply using microvascular anastomosis. Cao *et al*. implemented this strategy by implanting a PLGA/β-TCP scaffold coated in rhBMP-2 into the latissimus dorsi of a primate for 3 weeks, and then orthotopically transplanting it into a mandibular defect (Figure 5) [26]. This showed success, with a bone volume fraction ratio of 85% and new bone of 60% [26]. The masseter muscle has also been studied as an *in vivo* bioreactor, where a PCL/β-TCP scaffold was prevascularized in the masseter muscle for two months before being implanted in a mandibular defect with a vascular pedicle [80]. New capillary formation origination from the facial artery was observed, along with enhanced bone formation. While these two studies were performed in animal models, there are also several case reports of living bioreactor studies performed in humans. Graft maturation in either the latissimus dorsi or gastrocolic omentum with subsequent implantation in the mandible restored masticatory function for two patients and lasted for several months, though the small sample size of this evidence should be noted [81, 82]. The downside to bioreactor pre-vascularization is that two surgeries are required.

The third approach is the incorporation of bioactive ions into scaffold materials. Silicate glasses release silicon, calcium, phosphorous, and sodium ions as they dissolve, which are osteogenic and promote neovascularization [83, 84]. The mechanism behind this is thought to be acting via both intracellular and extracellular responses and includes gene upregulation and increased release of vascular endothelial growth factor and basic fibroblast growth factor [83]. Specifically, silicon has been shown to upregulate the expression of the transcription factor NRF2 which results in protection against oxidative stress and enhances osteogenic capacity [85]. The chemical composition of bioactive glasses can be varied to tailor their rate of biodegradation. There is limited evidence *in vivo*, but bioactive glasses have been shown to encourage vascularization in a few studies [84]. The effect of bioactive ions on bone regeneration has been studied in mandibular defect models; however, there is no existing work on their effect on the vascularization of mandibular defects, thus creating an area of opportunity.

While these three approaches show promise, there is still a major hurdle in the area of vascularization. In most studies, researchers evaluate the presence and size of blood vessels, but there is a lack of functionality assessments for how well the vessels actually perfuse the construct [19]. In summary, the vascularization of scaffolds is a major challenge for mandibular tissue engineering but creates opportunities for innovation through a variety of approaches.

## Innervation

In the field of bone tissue engineering, more focus has been given to the importance of angiogenesis; however, innervation is often overlooked, despite the presence of nerves in the periosteum and bone marrow. The inferior alveolar nerve innervates most of the maxillofacial region but is often removed during mandibular resection. This can leave patients with loss of sensation in the lips and chin, and therefore trouble chewing and speaking. Not only does the loss of this nerve impact the patient functionally, but it has also been shown that nerves play a role in the bone regeneration process itself. Neuropeptides and neurotransmitters are expressed in the healing microenvironment and contribute to the regulation of bone repair [86]. Schwann cells release paracrine factors that play an active role in tissue repair [86]. The role of nerves has been studied specifically in mandibular repair. In a rabbit model of mandibular distraction osteogenesis, resection of the alveolar nerve led to significantly lower bone mineral density, bone mineral volume, and trabeculae thickness and organization [87]. In another study, when an osteotomy was made in the mandible of a mouse who had had the inferior alveolar nerve resected, it resulted in a lack of cellular proliferation and impaired defect closure [86]. These studies highlight the importance of adequate inferior alveolar nerve innervation for both function and bone regeneration.

**Figure 6.**
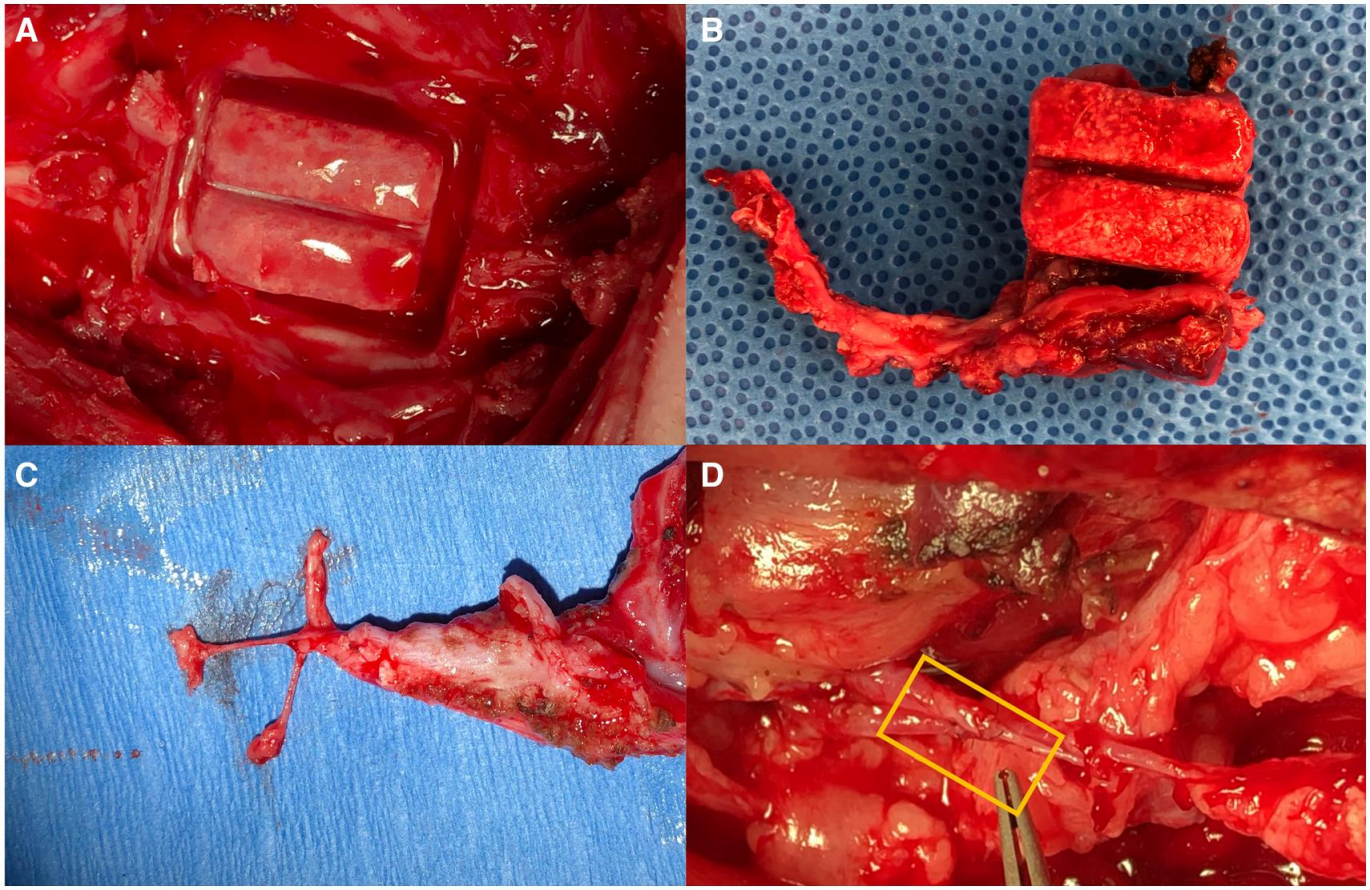
(**A**) Bioreactor in place on the periosteum prior to harvest, (**B**) newly formed bone with central channel, (**C**) nerve, artery and vein within pedicle attached to harvested tissue, (**D**) anastomosis (within boxed region) connecting flap circulation to systemic circulation (reproduced with permission from [89]).

Currently, there is a lack of bone tissue engineering solutions that can accurately maintain or recapitulate the nerve components. Although this is an important issue in mandibular tissue engineering, in a review by Dalfino *et al*. none of the included studies mentioned an approach for scaffold innervation. However, a few attempts at addressing this challenge have been made. For the scenario where the inferior alveolar nerve is not intact, Ye *et al.* designed a titanium implant coated with nerve growth factor (NGF), chondroitin sulfate and HA [88]. In a canine mandibular defect model, upregulation of NGF, neurogenic differentiation and osteogenic differentiation genes was observed [88]. For the scenario where the inferior alveolar nerve is still intact, Watson *et al*. developed a complex bioreactor that includes a central protuberance to allow for a channel within the regenerated bony tissue where a neurovascular bundle can run (Figure 6) [89]. This channel allows for correct anatomical positioning of the inferior alveolar nerve while minimizing risk for nerve injury [90]. This resulted in a bone volume fraction of 64.5% and the presence of viable osteoblasts within the tissue [89].

It is clear that the presence of the inferior alveolar nerve is crucial not only for preserving the patient’s functional status but also for the capacity of a mandible defect to repair itself. This is a very promising area of future research in mandibular tissue engineering.

## Personalization

While current technologies are able to restore much of the mandibular form and function to patients, there is still room for them to be further personalized. The future of mandibular tissue engineering scaffolds will be implants that are patient-specific, both in shape and biological components.

Personalizing the shape of scaffolds for mandibular repair can greatly help restore patients’ self-image by using the unaffected area of the mandible as a mirroring guide. This will rely largely on computer digital technology and the fidelity of 3D printers. For example, Mohammed *et al.* used a patient CT scan to create a model of the mandible and then translated the healthy segment to the defect area by mirroring. Medical grade titanium was then printed by SLS using this model, which resulted in an excellent model of the patient’s anatomy [91]. Another possibility for custom-shaped scaffolds is using an injectable, shape-adapting hydrogel that gels *in situ* after being filled into a bone defect. However, it is difficult to have precise control over the flow of the hydrogel due to gravity, therefore it may be advantageous to combine rigid 3D scaffolds with hydrogels to overcome this limitation [67].

The benefit of personalizing the biological component of these constructs is minimizing the possibility of an immune response to non-autologous cells. Allogeneic cells have the possibility of stimulating a foreign body response that will result in fibrotic tissue formation around the implant, ultimately compromising its ability to function. The first study using autologous mesenchymal stem cells and a biodegradable scaffold that was analogous to the clinical setting was performed in 2004, where BMSCs were harvested from the porcine ilium, seeded on a PLGA scaffold, and then implanted in a full-thickness mandibular defect [92].

**Figure 7.**
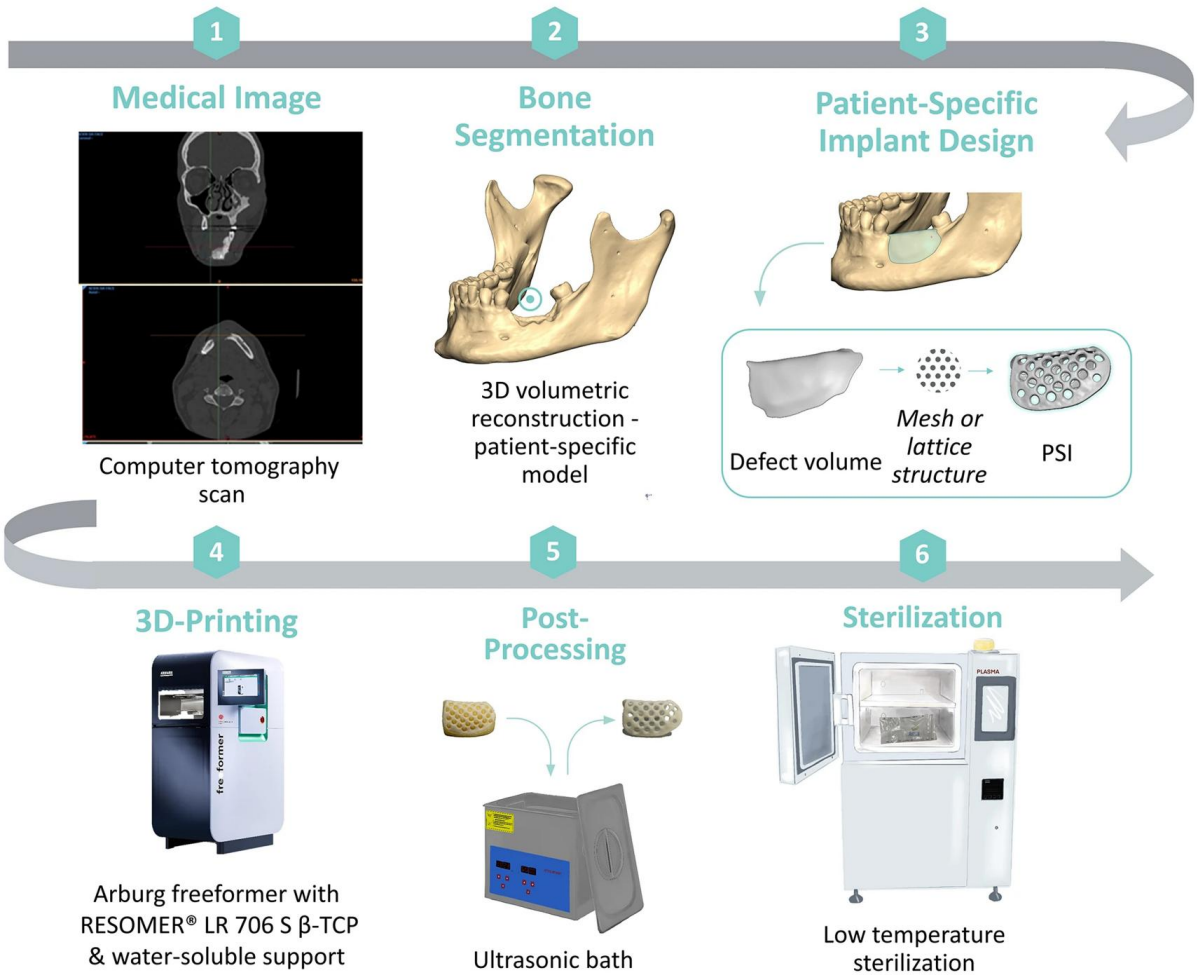
Process diagram for fabrication of personalized mandibular implant. The patient’s CT scan is used to create a 3D image, which enables the design of a customized reconstructive scaffold. The scaffold model is then 3D-printed, processed and sterilized so that it is ready for implantation into the same patient (reproduced with permission from [93]).

Translating fully personalized implants to clinical use will require investment in resources and personnel (Figure 7).

Ideally, one can envision a process where the patient comes in prior to mandibular resection and undergoes a procedure to harvest autologous stem cells. These cells will be cultured in a good manufacturing practice (GMP) level facility and tested for sterility and phenotype. At the same time, an implant designed from patient scans will also be manufactured in the GMP facility and the cells can subsequently be seeded on it. When the patient returns, mandibular resection can be performed, and the patient-specific construct will be ready for implantation. A close attempt at this was conducted in 2013 by Wolff *et al*. In three human patients with ameloblastomas, 3D patient images were used to design reconstruction plates that were printed out of titanium using SLS. ASCs were obtained from the patient’s abdominal walls and expanded in the presence of β-TCP and rhBMP-2 in a GMP clean room. ASCs and the reconstruction plates were placed in the defects and the process was found to successfully bridge the gap [94].

## Controlled release

The next area of opportunity in the field of mandibular tissue engineering is controlled release. While progress has been made in the basic incorporation of biological factors into tissue-engineered scaffolds, there is still a need for better spatiotemporal control over how the factors are released. The aim should be to mimic the complexity of natural tissue by releasing biological factors, and having fine control over this will enhance the quality and speed of tissue repair and regeneration. Drugs and other molecules have known short half-lives *in vivo*, so it is important to balance delivering an effective dose at the defect site while at the same time not overdosing and causing negative side effects.

Drugs and biological factors can be incorporated into scaffolds via physical or chemical methods, including adsorption onto the scaffold surface, direct incorporation into the scaffold material, or encapsulation in a carrier within the scaffold. Factor release can occur by diffusion or as a result of degradation of the scaffold. The release profile can be adjusted by altering the scaffold material composition, the delivery mechanism, or the dose of the biological factor. At the same time, it is imperative to not negatively affect the structural or mechanical properties of the scaffold and to not damage or release too quickly the biological factor itself [95]. The inability to control release characteristics can necessitate the use of excessive dosages of biological factors, which can have unwanted side effects for patients [96].

There are multiple approaches to controlled release for bone tissue engineering. First is incorporating the biological factor into a hydrogel matrix that will release the factor as it is degraded. Due to their relatively fast degradation rates, hydrogels often have a burst release within minutes followed by the sustained release that lasts for up to 48 hours, making this not an ideal system for long-term release [95, 97–99]. However, the polymer content and degree of polymer crosslinking can be manipulated to prolong hydrogel degradation and extend the time of factor release. A second approach is the use of micro or nanosphere carriers. The drug or biological factor can either be adsorbed to the carrier surface or encapsulated within the sphere itself. Adsorption will result in a burst release, while encapsulation will result in an initial burst release followed by a sustained release, and either method may be desirable depending on the biological factor being incorporated. The spheres can be made of polymers or hydrogels and can be solid, porous, or fibrous [95]. A drawback to the carrier method is that the spheres can migrate away from the defect site, so they are often used in combination with a structural scaffold to limit migration. Finally, porous scaffolds can mediate biological factor delivery. These could include hyaluronic acid, chitosan, alginate, collagen, fibrin, and many other naturally occurring scaffold materials [95]. The scaffolds can be further modified by coating with specific or nonspecific binding substrates for the molecule, such as heparin, to better control release from the scaffold.

**Figure 8.**
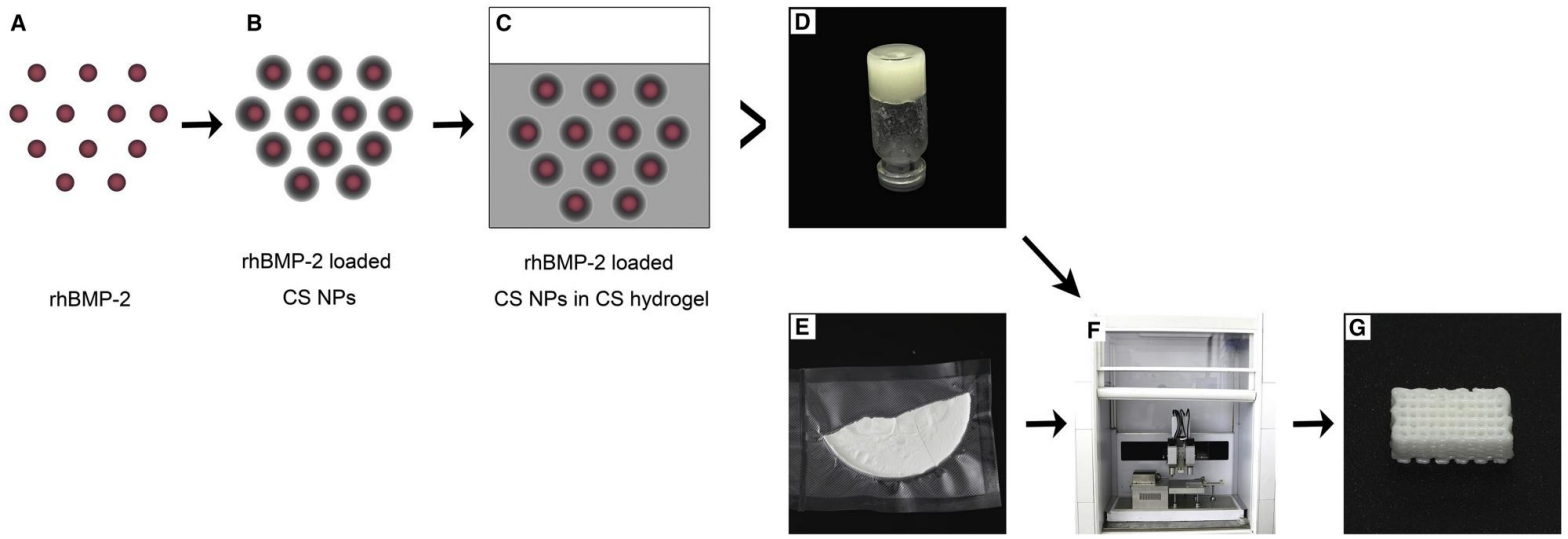
Preparation of PLGA/nano hydroxyapatite/chitosan/rhBMP-2 nanocarrier-scaffold complex (**A**–**D**). Lyophilized scaffold material (**E**). 3D bioprinter and final scaffold product (**F**, **G**) (reproduced with permission from [25]).

There has been some initial research on controlled release specifically mandibular tissue engineering, all focused on BMP-2. Gas-foamed PLA has been loaded with low and high doses of rhBMP-2, resulting in both dosages having an initial burst release followed by a slow release for 24 days. When placed into a mandibular ramus defect model in rats, the high dosage resulted in the greatest degree of bone replacement. It is hypothesized that the low dosage group did not form as much new bone due to the polymer degradation forming an acidic environment over time that the small amount of rhBMP-2 was not able to counteract [96]. BMP-2 has also been loaded in a GelMA hydrogel and implanted in a rat mandible critical size defect. The incorporation of BMP-2 did not alter the degradation time or porous structure of the hydrogel and promoted bone regeneration and enhanced intramembranous ossification [100]. In another example of the hydrogel approach, non-glycosylated rhBMP-2 was added to hydrogel sponges made of poly(ethylene glycol) (PEG), collagen/HA (Col/HA) or PEG+Col/HA. Non-glycosylated rhBMP-2 was chosen because it is less soluble than its glycosylated counterpart, thereby causing it to release more slowly and minimizing diffusion. Release study results showed that PEG only had the highest amount of burst release on the first day while the PEG+Col/HA had the lowest, possibly because the released rhBMP-2 also adsorbed onto the high surface area of Col/HA in addition to diffusing out of the hydrogel. An *in vivo* dose response study showed that mature bone formation in a rat mandibular defect model was dependent on the rhBMP-2 dose loaded [101]. An example of the carrier approach is the use of chitosan/rhBMP-2 nanocarriers within a PLGA/nano HA scaffold (Figure 8).

After incubation in phosphate buffered saline for 48 hours only about 10% of the drug was released and after 30 days approximately 61% had been released, owing to the slow degradation of the chitosan hydrogel. In a rabbit mandibular defect model, the sustained release mechanism promoted mature new bone formation for up to 12 weeks [25].

Improved control over biological factor release kinetics is the future of mandibular tissue engineering. There are a variety of methods to incorporate drugs into tissue engineering scaffolds and tune their release over time. Ideally this strategy could be expanded to enable sequential or simultaneous delivery of multiple factors, in order to replicate the natural bone repair process in the mandible as closely as possible.

## Conclusion

Mandibular defects can arise from a variety of congenital and acquired etiologies and can leave people with significant impairments in speaking, eating, and swallowing, in addition to influencing their self-image and mental health. Prior to the advent of tissue engineering, the standard of care for mandibular reconstruction has been autografts, allografts, metal implants, and distraction osteogenesis. While these approaches can restore the form and function of the mandible at a very basic level, they lack the ability to fine-tune and optimize bone regeneration. The field of tissue engineering was created in part to address these challenges and is currently being developed for mandibular repair applications. Synthetic polymers or ECM-derived scaffolds can be manufactured through a variety of techniques with precise control over their structural properties. Stem cells from bone or non-bone sources can be pre-seeded and promote early bone regeneration, and biological factors such as BMP, TGF-β, and antimicrobials can further encourage cell adhesion and proliferation. While great progress has been made with tissue-engineered constructs, there is still room for improvement. The future of mandibular tissue engineering will require restoration of the inferior alveolar artery and nerve, personalization of the shape and biological components of scaffolds, and spatiotemporal control over drug release from the scaffold. With these goals in mind, tissue engineers will move closer to a bio-inspired, patient-specific, biomaterial that will regenerate bone that is structurally and biologically indistinguishable from native mandibular tissue, and will restore both form and function to patients.
